# Simultaneous Integrated Boost for Dose Escalation in Node-Positive Cervical Cancer: 5-Year Experience in a Single Institution

**DOI:** 10.3390/cancers15184647

**Published:** 2023-09-20

**Authors:** Elki Sze-Nga Cheung, Frederick Chun-Him Law, Nelson Tsz-Cheong Fung, Inda Sung Soong, Rico Hing-Ming Hung, Teddy Ka-Ho Tse, Ken Ka-Shing Wong, Philip Yuguang Wu

**Affiliations:** 1 Department of Clinical Oncology, Pamela Youde Nethersole Eastern Hospital, Hong Kong, China; 2 Department of Medical Physics, Pamela Youde Nethersole Eastern Hospital, Hong Kong, China

**Keywords:** cervical cancer, radiotherapy, volumetric modulated arc therapy, simultaneous integrated boost, image-guided brachytherapy, nodal control

## Abstract

**Simple Summary:**

Nodal control is a major challenge for locally advanced cervical cancer (LACC) treated with definitive chemoradiotherapy. The optimal radiotherapy regime for patients with node-positive disease is yet to be defined. Modern image-guided intensity-modulated radiotherapy offers the potential for dose escalation to involved nodes while minimizing doses to organs at risk. This study reports the efficacy and toxicity of a simultaneous integrated boost in an Asian cohort with node-positive LACC in the context of contemporary volumetric modulated arc therapy and magnetic resonance image-guided adaptive brachytherapy. A total of 234 involved nodes in 54 patients were analyzed. Excellent nodal control was achieved, with four (2%) boost-volume failures occurring in three patients. The 2-year actuarial regional nodal control (RNC), pelvic control (PC), locoregional control (LRC), disease free survival (DFS), and overall survival (OS) were 93%, 87%, 87%, 78%, and 85%, respectively. The incidence of grade ≥ 3 radiotherapy-related toxicity was low. The 5-year local experience demonstrated excellent treatment outcomes with an acceptable toxicity profile.

**Abstract:**

This study retrospectively evaluates clinical outcomes of dose escalation to involved nodes using volumetric modulated arc therapy (VMAT) with simultaneous integrated boost (SIB) for node-positive locally advanced cervical cancer (LACC) at a single institution. Consecutive patients with node-positive LACC (FIGO_2018_ IIIC1-IVA) who received definitive chemoradiotherapy by VMAT 45 Gy in 25 fractions with SIB to 55–57.5 Gy, followed by magnetic resonance image-guided adaptive brachytherapy (IGABT) between 2018 and 2022 were identified. A standardized strategy regarding nodal boost delivery and elective para-aortic (PAO) irradiation was employed. Primary endpoints were involved nodal control (INC) and regional nodal control (RNC). Secondary endpoints were pelvic control (PC), locoregional control (LRC), disease-free survival (DFS), overall survival (OS), failure pattern, and radiotherapy-related toxicities. A total of 234 involved nodes (182 pelvic and 52 PAO) in 54 patients, with a median of 3 involved nodes per patient (range 1–16), were analyzed. After a median follow-up of 19.6 months, excellent INC was achieved, with four (2%) boost-volume failures occurring in three patients. The 2-year actuarial RNC, PC, LRC, DFS, and OS were 93%, 87%, 87%, 78%, and 85%, respectively. Adenocarcinoma histology was associated with worse RNC (*p* = 0.02) and OS (*p* = 0.04), whereas the primary tumor maximum standardized uptake value (SUVmax) was associated with worse PC (*p* = 0.04) and LRC (*p* = 0.046) on univariate analysis. The incidence of grade ≥3 acute and late radiotherapy-related toxicity were 2% and 4%, respectively. Treatment of node-positive LACC with VMAT with SIB allows safe and effective dose escalation. The 5-year local experience demonstrated excellent treatment outcomes without additional toxicity.

## 1. Introduction

Globally, cervical cancer is the fourth most frequent cancer in women with an age-standardized incidence of 13.3 cases per 100,000 women-years and mortality rate of 7.2 deaths per 100,000 women-years [1]. In Hong Kong, cervical cancer ranked the seventh most common cancer in women in 2020, with a crude incidence rate of 13.7 per 100,000 women [2].

For patients with locally advanced cervical cancer (LACC), definitive treatment involves external beam radiotherapy (EBRT) with concurrent chemotherapy followed by brachytherapy. Over the past decade, the standard of care has evolved from three-dimensional conformal radiation therapy to intensity-modulated radiation therapy for external beam radiotherapy and from a historical point-based two-dimensional approach to a three-dimensional image-guided adaptive approach for brachytherapy (IGABT). In particular, IGABT allows dose escalation to produce excellent local control and survival while minimizing radiotherapy-related toxicity and preserving quality of life [3]. The improvement of clinical outcomes when using IGABT is impressive [3,4,5]. Three-year local and pelvic control rates are reported in the ranges of 91–95% and 84–93%, respectively [3,6,7,8,9].

In this modern era of IGABT with well-controlled primary tumors, regional nodal and distant metastasis have become the predominant forms of failure and major challenges for cures [10]. Within the EMBRACE I study cohort, with 1338 patients analyzed, the nodal control rate at 3 years was 92% in the node-negative group, and 82% in the node-positive group, i.e., an absolute difference of 10%. While the involved nodes at the time of diagnosis were mainly located in the pelvis, nodal failures were most often reported in the para-aortic (PAO) region [11]. Furthermore, it is well recognized that patients with node-positive LACC are at higher risk of all types of failure [10,11].

Treatment intensification to sterilize involved nodes and treat subclinical PAO metastasis has, therefore, been explored to further improve outcomes for node-positive LACC. Some studies advocate surgical debulking of bulky lymph nodes prior to chemoradiotherapy to improve the chance of complete sterilization [12,13]. On the other hand, in the era of modern radiotherapy technology and treatment precision, there is increasing interest in definitive chemoradiotherapy with dose escalation to involved nodes, in the form of sequential or simultaneous integrated boost (SIB) [14,15,16,17]. SIB is attractive for its potential advantage in superior conformality and control of doses to organs at risk (OAR) without increasing OTT. However, the optimal radiotherapy dose prescription remains controversial, and the dose–response relationship for nodal control has not been clearly defined [16,18,19].

Accurate nodal staging and detection of PAO metastasis can be challenging. Even with positron emission tomography–computed tomography (PET-CT), the false negative rate for PAO lymph node involvement is 20–25% in patients with pelvic nodal disease [20,21]. The use of surgical PAO staging to define the extent of nodal disease and tailor the radiotherapy portal has been practiced in some parts of the world [16,17,18,19], but with concerns about morbidity, prolonged OTT, and uncertain impact on the overall outcome. Alternatively, elective PAO irradiation for high-risk patients is widely adopted to eradicate subclinical disease in the PAO region below the detection threshold of imaging [18,22]. Although no consensus in patient selection for elective PAO irradiation exists, the most commonly considered risk factors include the number of positive pelvic nodes and the presence of common iliac nodes [23,24,25]. Other factors such as metabolic activity on PET imaging, and primary tumor bulk have also been reported [19,23]. Individual retrospective studies have reported low rates of PAO failure and acceptable toxicity with elective PAO irradiation in the context of modern IMRT [18,22].

Due to the lack of high-quality prospective evidence on the optimal strategy, the management of clinically positive nodes is considerably heterogeneous across the world, ranging from nodal surgery to a variety of prescription practices for nodal boost and elective PAO irradiation [4]. With evolving clinical evidence and the adoption of volumetric modulated arc therapy (VMAT) internationally, our institution has implemented a standard strategy for VMAT delivery of SIB to all patients with node-positive LACC, and elective PAO irradiation for high-risk subgroups since 2018. The purpose of this study is to report on our 5-year local experience with this approach, including oncological outcomes and treatment-related toxicity.

## 2. Materials and Methods

### 2.1. Study Design

Consecutive patients with histologically confirmed International Federation of Gynaecology and Obstetrics 2018 (FIGO_2018_) [26] stage IIIC1-IVA node-positive LACC who received definitive VMAT 45 Gy in 25 fractions with SIB, with or without concurrent chemotherapy, followed by IGABT at the Department of Clinical Oncology of Pamela Youde Nethersole Eastern Hospital from 1 January 2018 to 31 December 2022 were retrospectively analyzed.

Data on patient demographics, clinicopathologic characteristics, radiotherapy treatment, and clinical outcomes were retrospectively collected from the clinical and radiotherapy treatment records. Patients were clinically staged according to FIGO 2018 [26] and the American Joint Committee on Cancer (AJCC) TNM staging version 9 [27]. Pre-treatment imaging included magnetic resonance imaging (MRI) of the pelvis and either contrast CT of the thorax, abdomen, and pelvis, or PET-CT. Radiological nodal involvement was defined as enlargement (>1 cm in short axis), presence of suspicious morphology, or positive uptake on PET-CT. Nodal surgery of any form was not allowed.

### 2.2. Treatment Delivery

#### 2.2.1. External Beam Radiotherapy

Patients were set-up in a supine position and immobilized in a customized vacuum bag. Simulation CT images with intravenous contrast and a slice thickness of 3 mm were acquired in both comfortably full- and empty-bladder to capture the range of internal motion of target volumes, and co-registered with MRI and PET-CT for contouring.

The primary tumor clinical target volume (CTV-T) included the primary gross tumor volume (GTV-T), remaining cervix, bilateral parametria, uterus, and 2 cm of uninvolved vagina from the most distal aspect of GTV-T. The CTV-T was defined in both the full and empty bladder phases before generating the internal target volume (ITV). The elective nodal clinical target volumes (CTV-E) consisted of the bilateral internal iliac, external iliac, obturator, presacral, and common iliac nodal regions up to the level of aortic bifurcation. CTV-E was extended to include the PAO region if there were 3 or more involved pelvic nodes, or any involved node at or above the common iliac region; and to include the inguinofemoral region if there were involved inguinal nodes or primary tumor involvement at the distal vagina. All clinically involved nodes were contoured individually and a 3 mm circumferential margin was applied to form the involved nodal clinical target volumes (CTV-N). The planning target volumes (PTV) were generated by applying a 5 mm margin to the respective CTVs.

The dose prescription was 45 Gy in 25 fractions over 5 weeks with SIB to 55 Gy (at 2.2 Gy per fraction) for nodes located inside the true pelvis, or 57.5 Gy (at 2.3 Gy per fraction) for nodes located outside the true pelvis, by VMAT. This resulted in a total dose of approximately 60 Gy EQD2_10_ to the involved nodes, considering dose contribution from BT inside the true pelvis [28]. The VMAT was delivered on Truebeam™ (Varian Medical Systems, Palo Alto, CA, USA) using 6 MV photon, with online cone beam CT verification complemented by 6-degrees-of-freedom couch corrections. The planning objectives and dose specifications for tumor targets and OAR were adapted from the EMBRACE II study protocol version 1.0 [29].

#### 2.2.2. Brachytherapy

After 5 weeks of EBRT, MR-based IGABT was delivered in four fractions over 2 weeks for all patients. Patients underwent two sessions of applicator implant, and two fractions were delivered on consecutive days after each implant. A combined intracavitary–interstitial (IC/IS) technique was used to achieve the planning aims where required. Imaging, target volume definition, applicator reconstruction, reporting of dose–volume parameters, dose prescription, and quality assurance followed the GEC-ESTRO recommendations [30,31,32]. The objective was to limit the overall treatment time (OTT) of EBRT and IGABT to no more than 50 calendar days.

### 2.3. Follow-Up

Patients received follow-up according to the usual institutional protocol. Clinical assessment was performed at 2 weeks and 12 weeks after completion of radiotherapy, then every 6 months until 5 years, and annually thereafter. The first post-treatment response assessment, performed at 12 weeks after completion of radiotherapy, consisted of a clinical examination with cervical biopsy, MRI pelvis, and contrast CT abdomen or PET-CT for cases with PAO involvement at diagnosis.

Treatment failures were detected by clinical examination, histological diagnosis, or surveillance imaging, and were classified as local (within the cervix, vagina, parametria or uterus), nodal (pelvic or PAO), or distant (lymph nodes beyond the PAO region or other distant sites).

Radiotherapy-related toxicities were reported according to the National Cancer Institute Common Terminology Criteria for Adverse Events (NCI-CTCAE) version 5.0 [33]. Acute toxicity was defined as that occurring during treatment or within 3 months after treatment, whereas late toxicity was defined as occurring more than 3 months after treatment.

### 2.4. Endpoints and Statistical Analysis

The primary endpoints were involved nodal control (INC) and regional nodal control (RNC). INC was defined as the absence of the nodal persistence or recurrence within the nodal boost PTV. RNC was defined as the absence of pelvic and PAO nodal persistence or recurrence.

The secondary endpoints were pelvic control (PC), locoregional control (LRC), disease-free survival (DFS), overall survival (OS), failure pattern, and radiotherapy-related toxicity. PC was defined as the absence of local or pelvic nodal disease. LRC was defined as the absence of local, pelvic, or PAO nodal disease. DFS was calculated from the date of diagnosis to the date of first failure related to cervical cancer, whereas OS was calculated from the date of diagnosis to death from any cause or last follow-up. Patients were censored at the time of last follow-up or death. Failure patterns at the time of first treatment failure were analyzed. The relationship of nodal failures with boost or elective nodal volumes were evaluated.

Statistical analysis was conducted on SPSS, version 22 (Chicago, IL, USA). Kaplan–Meier estimates on the probability of clinical outcomes at 12, 18, 24, and 36 months were derived. The associations between clinical outcomes and relevant clinicopathological and treatment variables were tested using a Cox proportional hazards regression model. A *p*-value of less than 0.05 indicates statistical significance.

## 3. Results

A total of 58 consecutive patients received treatment in the study period. Four patients were lost to follow-up before the first post-treatment assessment at 3 months, leaving 54 patients with a total of 234 involved nodes for analysis. Of note, 11 patients were also recruited under the EMBRACE II study [19].

### 3.1. Patient Characteristics

The median age at diagnosis was 56 (range 27–80) years. The majority of patients had an ECOG performance status of 0 or 1 (93%) and Charlson comorbidity index (CCI) [34] of 0 to 2 (81%). Forty-six (85%) patients had squamous cell carcinomas, six (11%) had adenocarcinomas and two (4%) had other histological types (poorly differentiated carcinoma; mixed small cell neuroendocrine and adenocarcinoma). Most patients (76%) underwent PET-CT as initial staging. The median tumor width at diagnosis was 64 mm (range 41–105 mm) and the median primary tumor maximal standardized uptake value (SUVmax) on PET-CT was 16.3 (range 5.8–61.9).

The median number of involved nodes per patient was three (range 1–16). Twenty three (43%) patients had 1–2 nodes, 14 (26%) had 3–5 nodes, 13 (24%) had 6–9 nodes, and 4 (7%) had 10 or more nodes. With respect to the nodal distribution, 33 (61%) patients had nodal disease limited to the pelvis, while 21 (39%) had PAO involvement. For patients with PAO involvement, the median number of PAO nodes was 2 (range 1–7), and all of them had concurrent pelvic nodes. See Table 1 for detailed information.

### 3.2. Individual Nodal Characteristics

There were 234 involved nodes for analysis, of which 182 (78%) were pelvic nodes and 52 (22%) were PAO nodes. The nodal volume was 3 cm^3^ or above in 30% of nodes. The median nodal SUVmax was 3.9 (range 1.3–46). Clustering was observed in 78 (33%) nodes. For detailed information see Table 2.

### 3.3. Treatment Characteristics

All patients completed definitive EBRT and MR-based IGABT as planned. The majority (87%) of patients received concurrent chemotherapy and 74% of them received five cycles in total. The remaining patients did not receive chemotherapy due to medical comorbidities or patient refusal.

Regarding EBRT coverage, 17 (31%) patients received RT to the pelvis only and 37 (69%) patients had extended field RT to include the PAO region with or without the inguinofemoral region. The intent of PAO irradiation was elective in 16 (43%) and therapeutic in 21 (57%) patients. Regarding brachytherapy, nearly all patients (98%) received combined IC/IS treatment. The median OTT from the initiation of EBRT to the completion of IGABT was 44 days (range 41–79 days), and 49 (91%) patients completed treatment within 50 days. The median dose to high-risk clinical target volume (HRCTV) D_90_ was 94.2 Gy (range 85.1–98.5 Gy). The median doses to D_2cc_ of bladder, rectum, sigmoid, and small bowel were 85.4 Gy, 65.8 Gy, 68.4 Gy, and 59.4 Gy, respectively. For detailed information, see Table 3.

### 3.4. Treatment Efficacy

After a median follow-up duration of 19.6 months (range 6.8–55.2 months), four lymph node failures within boost volume (out of total 234 boosted nodes) were detected in three patients. All were located in the pelvis. For detailed information, see Table 4.

**Figure 1:**
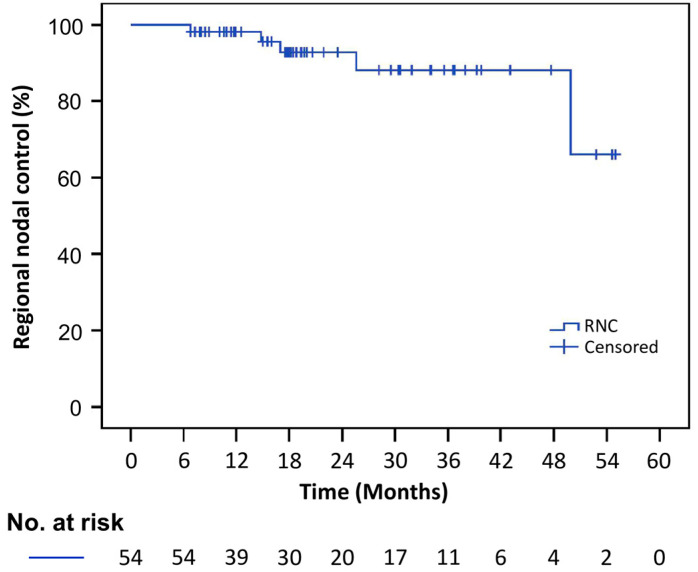
Kaplan–Meier estimates for regional nodal control.

A total of five patients experienced regional nodal failure. Table 5 presents a detailed analysis of events for each patient. Regional nodal failures were classified into (1) outside of radiation field, (2) inside of radiation field but outside of boost volume, or (3) inside of boost volume. A Kaplan–Meier plot for RNC is shown in Figure 1.

The 2-year actuarial INC, RNC, PC, LRC, DFS, and OS were 99%, 93%, 87%, 87%, 78%, and 85%, respectively. Adenocarcinoma histology was associated with significantly worse RNC (*p* = 0.02) and OS (*p* = 0.04) on univariate analysis. A high primary tumor SUVmax had a significant impact on PC (*p* = 0.04) and LRC (*p* = 0.046). The effect of the width of the primary tumor on PC was apparent but not statistically significant (*p* = 0.054). No significant impact on disease control or survival was detected in relation to the total number of nodes involved, nodal size, nodal volume, nodal distribution, and OTT. Table 6 summarizes the actuarial estimates for oncological outcomes. Table 7, Table 8 and Table 9 summarize the effect of clinicopathological and treatment variables on RNC, PC, LRC, DFS, and OS.

### 3.5. Failure Patterns

**Figure 2:**
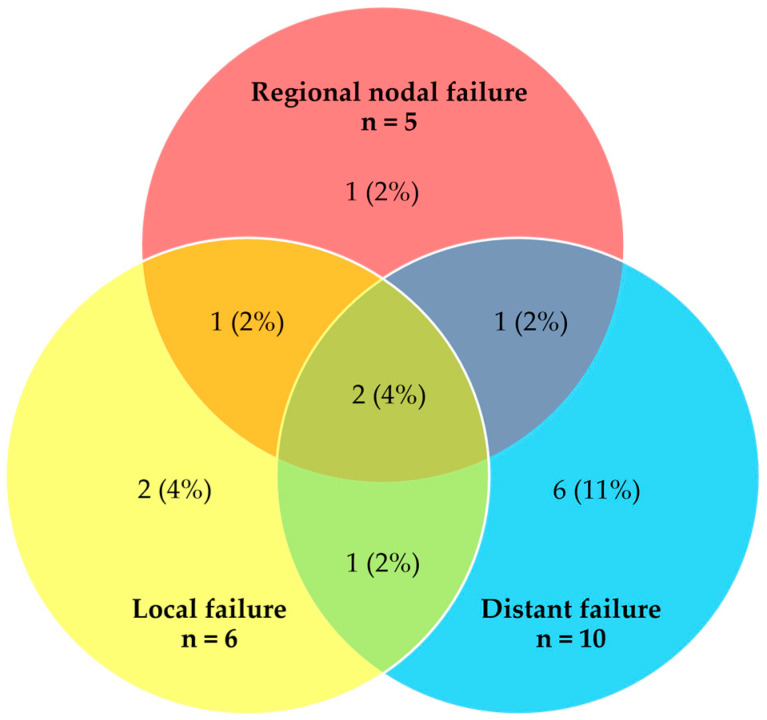
Venn diagram showing crude number and distribution of failures at the time of first relapse (n = 21 events, 14 patients).

Among the 54 patients analyzed, 40 patients (74%) remained in complete remission during follow-up. A total of 21 failure events occurred among 14 patients. Figure 2 illustrates the patterns of failure at the time of first relapse and the number of patients experiencing each failure type.

The predominant type of failure was distant with or without concurrent local and/or regional nodal failure (10 out of the total 14 patients who experienced any treatment failure). The median time to any treatment failure was 18.0 months (range 3.9–54.6 months). For the four patients who had locoregional-only failure, subsequent treatment included chemotherapy and SBRT. None of these patients were deemed suitable for salvage surgery. The majority of patients with distant failure (70%) received subsequent palliative chemotherapy.

### 3.6. Toxicity

Thirty-three percent of patients developed any grade 2 or above acute radiotherapy-related gastrointestinal (GI) or genitourinary (GU) toxicity. These were mostly GI toxicity, presenting as vomiting or diarrhea during chemoradiotherapy (29%). Only one patient (2%) developed grade 3 diarrhea with electrolyte disturbance and acute kidney injury requiring parenteral fluids.

Grade 2 or above late GU or GI toxicity occurred in five patients (10%), of which only two patients (4%) experienced grade 3 complications. One patient experienced grade 3 radiation proctitis. Another patient had grade 3 radiation-related ureteric stricture requiring stenting. The radiotherapy plans were reviewed and the affected organs were outside of the nodal boost PTV for both cases. No grade 4 or above radiotherapy-related toxicity occurred. For detailed information, see Table 10.

## 4. Discussion

The present study reported excellent treatment outcomes and favorable toxicity profiles in patients with node-positive LACC treated under a highly standardized treatment strategy involving dose escalation to involved nodes by SIB, and elective PAO irradiation. To our knowledge, this is the first publication to address the efficacy and toxicity of SIB for Asian patients with LACC in the context of contemporary image-guided VMAT planning and MR-based IGABT, and the results serve as a benchmark in real-world clinical evidence.

With the paradigm shift towards MR-based IGABT in the past decade achieving excellent local control, there is a pressing demand to address regional and systemic disease control, especially in patients with node-positive disease [10]. One of the attractive strategies is dose escalation to involved nodes, which has demonstrated encouraging outcomes [18,35,36,37]. However, the dose–response relationship for nodal control has not been clearly defined, thus consensus on the optimal dose prescription is lacking. In clinical practice, SIB is commonly prescribed in the range of 55–60 Gy in 25 fractions [18,36,37]. Bacorro et al. [38] suggested that increasing the total nodal dose from 50 Gy to 60 Gy EQD2 could significantly improve nodal control, especially for bulky nodes. Doses exceeding 60 Gy would raise concerns of increasing morbidity especially when applied in the PAO region, and data on its safety is scarce [39]. From retrospective series, it was also suggested that the dose–response relationship of involved nodes appears to be flat at 55 Gy to 60 Gy, thus the benefit of further escalation to above 60 Gy EQD2 is doubtful [28]. Among the limited evidence for nodal SIB in the context of chemoradiotherapy with IGABT, effective nodal control of 83% to 100% using median nodal doses of 55 Gy to 57.5 Gy has been reported [18,35,37]. It is noteworthy that for many of these studies, the overall regional nodal control was reported instead of the tumoricidal effect of SIB on individual nodes. In the present study, we observed excellent crude INC of 98% (4 failures out of 234 boosted nodes). A comparable series to ours by Jethwa et al. [37] utilized a similar technique of VMAT with SIB with a median prescription dose of 56.25 Gy in 25 fractions, and crude nodal control of 95% at 30 months was achieved.

From the prior literature, the control of involved nodes is adversely influenced by factors such as nodal volume (e.g., greater than 3 cm^3^), size larger than 2 cm, and prescription dose of less than 58 Gy in EQD2_10_ [35,38]. Another study reported a significant correlation between SUVmax and treatment failure in boosted nodes, and thus, intensification of treatment of nodes with a high SUVmax has been suggested [40]. It is worth noting that in our study, 30% of nodes were bulky and had a volume of more than 3 cm^3^. These bulky nodes were successfully controlled by SIB. The low nodal boost failure rate in our study did not allow evaluation of INC by risk factor, but this also suggests that our current SIB dose of 55–57.5 Gy in 25 fractions (i.e., approximately 60 Gy in EQD2_10_) is sufficient for the majority of patients.

Predictive and prognostic factors for locoregional control and survival identified in previous studies include tumor histology, stage, tumor size, nodal size, number, and presence of PAO nodes [10]. In our present study, we observed very encouraging 2-year actuarial RNC, LRC, DFS, and OS of 93%, 87%, 78%, and 85%, respectively, after a median follow-up of 19.6 months. Excellent disease control was achieved despite our local cohort consisting of a significant proportion of women with a high risk profile −39% of patients had FIGO stage IIIC2, 46% had common iliac involvement, 57% had ≥3 nodes, 31% had ≥6 nodes, and the median primary tumor width was 64 mm. Adenocarcinoma histology was significantly associated with worse RNC and OS, which is consistent with prior experience [10]. Moreover, the primary tumor SUVmax was associated with worse LRC and PC. This concurs with previous publications suggesting that a high primary tumor SUVmax may be predictive of poor response to radiotherapy and associated with inferior survival [41].

Different strategies have been developed to address micro-metastasis in the PAO region. One involves pre-therapeutic surgical nodal staging to identify subclinical nodal involvement, followed by tailored chemoradiotherapy, with the advantage of avoiding unnecessary PAO irradiation. Surgical staging could be combined with debulking of macroscopic nodes to increase the chance of complete sterilization by chemoradiotherapy. While ample evidence on the outcomes from different surgical approaches exists in early stage cervical cancer [42,43], the role of nodal surgery in the setting of locally advanced disease is less certain. Complications related to nodal surgery vary with surgical approach (transperitoneal, retroperitoneal, open, or laparoscopic) and include ureteral and vascular injury, lymphocyst, etc., with overall incidence reported to be between 7 and 10% [44,45,46]. On the other hand, the treatment landscape has shifted towards the use of elective PAO irradiation to high-risk patients based on radiological staging, to avoid surgical morbidity and delay in OTT. With increasing adoption of PET imaging with higher sensitivity and specificity for nodal staging compared to CT or MRI, as well as modern IMRT with a favorable toxicity profile, surgical nodal staging prior to definitive chemoradiotherapy is falling out of favor. In our cohort, 76% of patients had PET-CT staging at initial diagnosis. Patients who were considered at high risk of PAO relapse (three or more pelvic nodes or presence of common iliac node) were given elective PAO irradiation. Among the 16 patients who received elective PAO irradiation, only 1 patient experienced recurrence in the PAO region (this was also accompanied by distant metastases in the lungs and cervical node at the time of failure). Given the very good RNC achieved with our current treatment approach, the value of surgical nodal staging is questionable. Two randomized controlled trials, PAROLA [47] and CQGOG0103 [48], are underway to further address these issues.

It is well recognized that IMRT produces superior dose conformality. Using VMAT with stringent planning aims and control of doses to OAR, coverage probability planning, and intensive image guidance, we attained a favorable toxicity profile despite a liberal approach in dose escalation, even in patients with a high nodal disease burden, and elective PAO irradiation. We observed a low incidence rate of acute and late grade 3 gastrointestinal or genitourinary (GU) toxicities, of 2% and 4%, respectively. These rates compare favorably to similar IMRT series involving nodal boost, the late toxicity of which ranges from 4% to 12% at 3 years [18,37,49]. Notably, in our cohort, there was no reported severe duodenal toxicity among the 21 patients receiving nodal boost in the PAO region.

The predominant failure pattern in our cohort is at distant sites, which is consistent with the findings in the large EMBRACE I cohort [4]. This calls for strategies to intensify treatment, or the exploration of alternative agents to improve systemic control. The role of adjuvant or neoadjuvant chemotherapy to definitive chemoradiotherapy or surgery were assessed in randomized trials, but with limited success [50,51,52]. The addition of an anti-VEGF agent such as bevacizumab to definitive chemoradiotherapy was found to be safe in a phase II trial, but the benefit is yet to be proven in a randomized trial [53]. On the other hand, immune checkpoint inhibition has demonstrated encouraging activity in cervical cancer. Pembrolizumab, an anti-programmed cell death-1 monoclonal antibody was shown to prolong overall survival when combined with chemotherapy (with or without bevacizumab) in the first-line metastatic setting. The role of pembrolizumab combined with standard chemoradiotherapy for LACC is being investigated in the phase III KEYNOTE-A18 trial [54], and the results are eagerly awaited. Moving forward, further research on molecular biomarkers such as circulating human papillomavirus DNA [55] and tumor metabolic response on advanced imaging [41] may shed light on cervix cancer biology and predictors of treatment response, and stratify patients for treatment intensification or de-intensification.

The current study was a single institutional retrospective study with limited patient numbers, a relatively short follow-up duration, and lack of patient-reported outcomes. The retrospective nature of this study could result in under-reporting of low-grade toxicity. Moreover, longer follow-up is required to verify long-term treatment outcomes and observe late toxicity. Nevertheless, it is well known that most treatment failures occur within the first 2 years for cervical carcinoma [11]. Despite these limitations, our study represents one of the few reports on nodal dose escalation involving consecutive patients treated under a standardized treatment protocol of contemporary VMAT planning, intensive image guidance and systematic use of MR-based IGABT. It is also one of the first publications reflecting real-world evidence in Asian patients, and in particular, of a relatively high risk disease profile. Given the very encouraging efficacy and safety outcomes achieved in our cohort, the adoption of a similar approach in radiological staging, dose escalation to involved nodes using VMAT SIB, and elective PAO irradiation should be considered in clinical practice for patients with node-positive LACC. Further validation from large prospective multicenter series is highly anticipated.

## 5. Conclusions

Treatment of node-positive LACC by VMAT with SIB allows safe and effective dose escalation. The 5-year institutional experience demonstrates encouraging treatment outcomes and a low incidence of severe treatment-related toxicity.

## Figures and Tables

**Table 1:** Clinicopathological characteristics (N = 54).

Age, median (range), y	56 (27–80)
ECOG, *n* (%)	
0 or 1	50 (93)
≥2	4 (7)
CCI, *n* (%)	
≤2	44 (81)
>2	10 (19)
Histology, *n* (%)	
SCC	46 (85)
Adenocarcinoma	6 (11)
Others ^1^	2 (4)
FIGO stage, *n* (%)	
IIIC1	31 (57)
IIIC2	21 (39)
IVA	2 (4)
TNM stage—T stage, *n* (%)	
T1b3	1 (2)
T2a	6 (11)
T2b	28 (52)
T3a	3 (6)
T3b	14 (26)
T4a	2 (4)
TNM stage—N stage, *n* (%)	
N1	33 (61)
N2	21 (39)
Width of primary tumor, median (range), mm	64 (41–105)
Parametrial involvement, *n* (%)	48 (89)
PET-CT staging, *n* (%)	41 (76)
Primary tumor SUVmax ^2^, median (range)	16.3 (5.8–61.9)
Maximal nodal short axis ^3^, median (range), mm	12 (6.3–49.0)
Total no. of involved LN, median (range)	3 (1–16)
1–2	23 (43)
3–5	14 (26)
6–9	13 (24)
≥10	4 (7)
Nodal distribution	
Pelvic—low pelvis	54 (100)
Pelvic—common iliac	25 (46)
PAO	21 (39)
Median follow-up (range), months	19.6 (6.8–55.2)

^1^ Other histology types include poorly differentiated carcinoma, and mixed small cell neuroendocrine and adenocarcinoma. ^2^ Among 41 patients with PET-CT performed. ^3^ Node with the largest dimension in short axis was selected as the representative node for analysis. Abbreviations: ECOG = Eastern Cooperative Oncology Group, CCI = Charlson comorbidity index, SCC = squamous cell carcinoma, ADC = adenocarcinoma LN = lymph node, PAO = para-aortic, SUVmax = maximum standardized uptake value

**Table 2:** Individual nodal characteristics (N = 234).

Total no. of pelvic LN, *n* (%)	182 (78)
Total no. of pelvic LN—low pelvic, *n* (%)	140 (60)
Total no. of pelvic LN—common iliac, *n* (%)	42 (18)
Total no. of PAO LN, *n* (%)	52 (22)
Nodal volume, cm^3^	
<3	164 (70)
3–5	29 (12)
>5	41 (18)
Nodal SUVmax ^1^, median (range)	3.9 (1.3–46)
Presence of clustering, *n* (%)	78 (33)

^1^ Among a total of 86 nodes with SUVmax data available. Abbreviations: LN = lymph node, PAO = para-aortic, SUVmax = maximum standardized uptake value.

**Table 3:** Treatment characteristics.

Concurrent chemotherapy ^1^	
Yes	47 (87)
No	7 (13)
EBRT coverage	
Pelvic	17 (31)
Pelvic + PAO	30 (56)
Pelvic + PAO + inguinofemoral	7 (13)
Intent of PAO irradiation ^2^	
Elective	16 (43)
Therapeutic	21 (57)
EBRT dosimetric parameters, median (range)	
GTV-T vol, cm^3^	132.1 (24.2–593.7)
ITV45 vol, cm^3^	1144.9 (730.3–2817.7)
Bladder V30, cm^3^	95.9 (75.9–100)
Bladder V40, cm^3^	64.8 (47.1–100)
Rectum V30, cm^3^	99.6 (65.9–100)
Rectum V40, cm^3^	90.8 (46.5–100)
Bowel V30, cm^3^	459.8 (156.7–1075.7)
Bowel V40, cm^3^	206.2 (56.2–558.0)
Conformality V36/PTV	1.50 (1.35–1.56)
Conformality V43/PTV	1.04 (0.98–1.10)
Brachytherapy technique	
IC + IS, *n* (%)	53 (98)
IC alone, *n* (%)	1 (2)
No. of IS needles loaded, median (range)	7 (0–17)
Brachytherapy dosimetric parameters, median (range), Gy	
GTV D_98_	99.4 (78.1–135.6)
HR CTV D_90_	94.2 (85.1–98.5)
IR CTV D_98_	60.9 (55.4–62.9)
D_2cc_ bladder	85.4 (63.7–94.7)
D_2cc_ rectum	65.8 (51.6–75.0)
D_2cc_ sigmoid	68.4 (49.9–78.9)
D_2cc_ small bowel	59.4 (44.2–78.7)
OTT, median (range), days	44 (41–79)
≤50, *n* (%)	49 (91)
>50, *n* (%)	5 (9)

^1^ Weekly cisplatin at 40 mg/m^2^, except in one patient with mixed histology (small cell carcinoma and adenocarcinoma) where a combination of etoposide and cisplatin was administered instead. ^2^ Among 37 patients who received PAO irradiation. Abbreviations: PAO = para-aortic; GTV-T = primary gross tumor volume; IC = intracavitary; IS = interstitial; GTV = gross tumor volume; HRCTV (high-risk clinical target volume); IRCTV (intermediate-risk clinical target volume); OTT = overall treatment time.

**Table 4:** Individual nodal failures within boost volume: characteristics and time to failure.

Patient	Location	Size in Short Axis (mm)	GTV-N Volume (cm^3^)	Nodal SUVmax	Clustering	Boost Dose (Gy)	Time to Failure (month)
1	External iliac	27	37.31	12	Yes	55	25.6
2	Internal iliac	9	0.59	2.6	No	55	17.1
3	Internal iliac	17	4.15	6	No	55	6.8
	External iliac	13	5.17	7.5	No	55	6.8

Abbreviations: GTV-N = nodal gross tumor volume; SUVmax = maximum standardized uptake value.

**Table 5:** Regional nodal failure: initial disease, treatment, and recurrence characteristics.

Histology	TNM Stage	Nodal Distribution at Diagnosis (no. of LN)	PAO Irradiation	Recurrence Characteristics			
				Failure Pattern	No. of Pelvic LN (In/Out-Field)	No. of PAO LN (In/Out-Field)	Subsequent Treatment
SCC	T2bN1M0	Pelvic (4)	Yes (Elective)	Pelvic nodal	1 (in-field)	0	SBRT
SCC	T2a2N2M0	Pelvic (1) + PAO (1)	Yes (Therapeutic)	Pelvic nodal + Local	1 (in-boost)	0	Chemo
SCC	T2bN1M0	Pelvic (3)	Yes (Elective)	PAO nodal + Distant	0	6 (in-field)	Palliative RT
ADC	T2bN1M0	Pelvic (1)	No	Pelvic nodal + PAO nodal + Local + Distant	5 (in-field) 1 (in-boost)	9 (out-field)	None
ADC	T2bN1M0	Pelvic (2)	No	Pelvic nodal + PAO nodal + Local + Distant	5 (in-field) 2 (in-boost)	1 (out-field)	Chemo

Abbreviations: SCC = squamous cell carcinoma, ADC = adenocarcinoma, LN = lymph node, PAO = para-aortic, SBRT = stereotactic body radiation therapy, Chemo = chemotherapy, RT = radiotherapy.

**Table 6:** Actuarial estimates for INC, RNC, PC, LRC, DFS, and OS.

	INC	RNC	PC	LRC	DFS	OS
12 months	99%	98%	94%	94%	88%	91%
18 months	99%	93%	87%	87%	78%	91%
24 months	99%	93%	87%	87%	78%	85%
36 months	97%	88%	82%	82%	74%	80%

**Table 7:** Regional nodal control (RNC) stratified by relevant clinicopathological and treatment variables.

		RNC	
Variable		HR (95% CI)	*p*
Histology	SCC	Ref	
	ADC	10.99 (1.51–79.88)	0.02
	Others ^1^	No event	
FIGO stage	IIIC1	Ref	
	IIIC2 or above	0.38 (0.04–3.49)	0.40
Concurrent chemotherapy	Yes	Ref	
	No	0.76 (0.08–7.56)	0.82
Nodal distribution	Pelvic only	Ref	
	Pelvic + PAO	0.42 (0.05–3.79)	0.44
Total no. of LN	1–2	Ref	
	≥3	0.51 (0.08–3.17)	0.47
Total nodal volume (cm^3^)	Continuous variable	1.00 (0.97–1.03)	1.00
Maximal nodal size in short axis (mm)	Continuous variable	1.00 (0.89–1.13)	0.94
Maximal nodal SUVmax ^2^	Continuous variable	1.02 (0.87–1.19)	0.79
Width of primary tumor (mm)	Continuous variable	1.01 (0.93–1.09)	0.83
GTV-T volume (cm^3^)	Continuous variable	1.00 (0.98–1.01)	0.66
Primary tumor SUVmax ^3^	Continuous variable	1.03 (0.93–1.14)	0.54
OTT	≤50 days	Ref	
	>50 days	No event	

^1^ Other histology types include poorly differentiated carcinoma, and mixed small cell neuroendocrine and adenocarcinoma. ^2^ Among 41 patients with PET-CT performed, node with the highest SUVmax value was selected as the representative node for analysis. ^3^ Among 41 patients with PET-CT performed. Abbreviations: SCC = squamous cell carcinoma; ADC = adenocarcinoma; LN = lymph node; PAO = para-aortic; GTV-T = primary gross tumor volume; SUVmax = maximum standardized uptake value; OTT = overall treatment time.

**Table 8:** Pelvic control (PC) and locoregional control (LRC) stratified by relevant clinicopathological and treatment variables.

		PC		LRC	
Variable		HR (95% CI)	*p*	HR (95% CI)	*p*
Histology	SCC	Ref		Ref	
	ADC	4.03 (0.77–20.99)	0.10	4.03 (0.77–20.99)	0.10
	Others ^1^	No event		No event	
FIGO stage	IIIC1	Ref		Ref	
	IIIC2 or above	1.27 (0.28–5.69)	0.76	0.94 (0.22–3.99)	0.94
Parametrial involvement	No	Ref		Ref	
	Yes	2.85 (0.55–14.74)	0.21	2.85 (0.55–14.74)	0.21
Concurrent chemotherapy	Yes	Ref		Ref	
	No	No event		0.52 (0.06–4.54)	0.55
Nodal distribution	Pelvic only	Ref		Ref	
	Pelvic + PAO	1.42 (0.32–6.34)	0.65	1.04 (0.25–4.40)	0.96
Total no. of LN	1–2	Ref		Ref	
	≥3	0.67 (0.15–3.00)	0.60	0.67 (0.15–3.00)	0.60
Total nodal volume (cm^3^)	Continuous variable	1.00 (0.98–1.03)	0.94	1.00 (0.98–1.03)	0.97
Maximal nodal size in short axis (mm)	Continuous variable	1.02 (0.94–1.11)	0.61	1.02 (0.93–1.10)	0.72
Maximal nodal SUVmax ^2^	Continuous variable	1.00 (0.86–1.17)	1.00	1.00 (0.85–1.17)	0.94
Width of primary tumor (mm)	Continuous variable	1.05 (1.00–1.11)	0.054	1.05 (1.00–1.10)	0.08
GTV–T volume (cm^3^)	Continuous variable	1.00 (1.00–1.01)	0.28	1.00 (1.00–1.01)	0.26
HR CTV D_90_	Continuous variable	0.91 (0.72–1.14)	0.39	0.94 (0.75–1.19)	0.61
Primary tumor SUVmax ^3^	Continuous variable	1.07 (1.00–1.15)	0.04	1.07 (1.00–1.14)	0.046
OTT	≤50 days	Ref		Ref	
	>50 days	No event		No event	

^1^ Other histology types include poorly differentiated carcinoma, and mixed small cell neuroendocrine and adenocarcinoma. ^2^ Among 41 patients with PET-CT performed, node with the highest SUVmax value was selected as the representative node for analysis. ^3^ Among 41 patients with PET-CT performed. Abbreviations: SCC = squamous cell carcinoma; ADC = adenocarcinoma; LN = lymph node; PAO = para-aortic; GTV-T = primary gross tumor volume; HRCTV = high-risk clinical target volume; SUVmax = maximum standardized uptake value; OTT = overall treatment time.

**Table 9:** Disease-free survival (DFS) and Overall survival (OS) stratified by relevant clinicopathological and treatment variables.

		DFS		OS	
Variable		HR (95% CI)	*p*	HR (95% CI)	HR (95% CI)
Age	Continuous variable	0.99 (0.94–1.04)	0.72	0.95 (0.89–1.02)	0.18
ECOG	0 or 1	ref		ref	
	≥2	0.97 (0.20–4.78)	0.98	1.64 (0.20–13.71)	0.65
CCI	0	ref		ref	
	1–2	0.63 (0.15–2.61)	0.52	0.50 (0.08– 3.01)	0.44
	≥3	1.24 (0.27–5.65)	0.78	0.90 (0.12–6.48)	0.92
Histology	SCC	ref		ref	
	ADC	2.30 (0.49–10.78)	0.29	6.07 (1.05–35.09)	0.04
	Others ^1^	1.61 (0.20–13.37)	0.66	4.31 (0.48–38.76)	0.19
FIGO stage	IIIC1	ref		ref	
	IIIC2 or above	1.95 (0.65–5.82)	0.23	2.37 (0.53–10.60)	0.26
Concurrent chemotherapy	Yes	ref		ref	
	No	0.97 (0.26–3.64)	0.96	0.79 (0.09–6.54)	0.82
Nodal distribution	Pelvic only	ref		ref	
	Pelvic + PAO	2.15 (0.72–6.42)	0.17	2.56 (0.57–11.46)	0.22
Total no. of LN	1–2	ref		ref	
	≥3	1.97 (0.61–6.38)	0.26	1.10 (0.25–4.90)	0.90
Total nodal volume (cm^3^)	Continuous variable	1.01 (1.00–1.03)	0.04	1.00 (0.99–1.02)	0.70
Maximal nodal size in short axis (mm)	Continuous variable	1.06 (0.99–1.12)	0.09	1.04 (0.97–1.11)	0.32
Maximal nodal SUVmax ^2^	Continuous variable	1.00 (0.90–1.11)	0.99	1.00 (0.83–1.19)	0.93
Width of primary tumor (mm)	Continuous variable	1.03 (0.99–1.08)	0.10	1.02 (0.96–1.09)	0.45
GTV-T volume (cm^3^)	Continuous variable	1.00 (1.00–1.01)	0.21	1.00 (0.99–1.01)	0.67
Primary tumor SUVmax ^3^	Continuous variable	1.04 (0.99–1.09)	0.13	1.04 (0.97–1.12)	0.23
OTT	≤50 days	ref		ref	
	>50 days	1.17 (0.15–9.19)	0.88	2.03 (0.24–17.02)	0.52

^1^ Other histology types include poorly differentiated carcinoma, and mixed small cell neuroendocrine and adenocarcinoma. ^2^ Among 41 patients with PET-CT performed, node with the highest SUVmax value was selected as the representative node for analysis. ^3^ Among 41 patients with PET-CT performed. Abbreviations: ECOG = Eastern Cooperative Oncology Group; CCI = Charlson comorbidity index; SCC = squamous cell carcinoma; ADC = adenocarcinoma; LN = lymph node; PAO = para-aortic; SUVmax = maximum standardized uptake value; GTV-T = primary gross tumor volume; OTT = overall treatment time.

**Table 10:** Radiotherapy-related toxicity.

	**Acute Toxicity**		
	**Any GU/GI**	**GU**	**GI**
Grade 2	31%	2%	29%
Grade 3	2%	0%	2%
	**Late Toxicity**		
	**Any GU/GI**	**GU**	**GI**
Grade 2	6%	2%	4%
Grade 3	4%	2%	2%

Remarks: no grade 4 or above toxicity was observed. Abbreviations: GU = genitourinary; GI = gastrointestinal.

## Data Availability

The data presented in this study are available on request from the corresponding author. The data are not publicly available due to patients’ privacy.
